# The regulation of long non-coding RNA 00958 (LINC00958) for oral squamous cell carcinoma (OSCC) cells death through absent in melanoma 2 (AIM2) depending on microRNA-4306 and Sirtuin1 (SIRT1) in vitro

**DOI:** 10.1080/21655979.2021.1955561

**Published:** 2021-08-12

**Authors:** Lei Jiang, Wenyu Ge, Yifei Cui, Xiaofeng Wang

**Affiliations:** a Department of Pathology, The Second Affiliated Hospital of Harbin Medical University, Heilongjiang. China; b Department of Stomatology, The Second Affiliated Hospital of Harbin Medical University. Harbin Institute of Technology, Heilongjiang Provincial Hospital, Heilongjiang, China; c Department of Pathology, Harbin Medical University Cancer Hospital, Heilongjiang, China; d Department of Stomatology, The Second Affiliated Hospital of Harbin Medical University, Heilongjiang, China

**Keywords:** LINC00958, miR-4306, AIM2, SIRT1, OSCC, p53

## Abstract

Long non-coding RNAs (lncRNAs) have been proposed as potential targets in OSCC gene therapy. Thus, the study aims to analyze how they exert functions in OSCC. LINC00958, AIM2, Gasdermin D (GSDMD) and tumor protein p53 (TP53) expression levels are analyzed by Quantitative Real-time PCR (qPCR) or Western blotting (WB) in OSCC cells lines. The roles of LINC00958 in cell proliferation, cell death, and GSDMD expression respectively were analyzed by Cell Counting Kit-8 (CCK8) assay, flow cytometry and Immunofluorescence (IF) assay. In addition, expressions of pyroptosis- and autophagy-related proteins are evaluated by WB detection. The targeted binding of LINC00958 and miR-4306 or AIM2 mRNA is predicted by bioinformatics analysis and detected by biodual luciferase system. RIP and qPCR assays analyze whether LINC00958 interacts with SIRT1. We found that LINC00958 showed upregulation in OSCC cells compared to normal oral epithelial cells. LINC00958 silencing significantly suppressed OSCC cell proliferation, induced cell death and reduced autophagy. LINC00958 regulated the levels of miR-4306 which binds to the 3ʹUTR of AIM2, and interacts with and modulates SIRT1 protein expression. LINC00958 regulated GSDMD and AIM2 levels, as well as p53 and SIRT1 levels. SIRT1 overexpression markedly reversed aforementioned effects of LINC00958. LINC00958 not only downregulated miR-4306 levels to activate the pyroptosis pathway mediated by AIM2 and promoted cancer cell survival but also induced a decrease in SIRT protein expression to further reduce p53 levels.

## Graphical abstract

**Figure:**
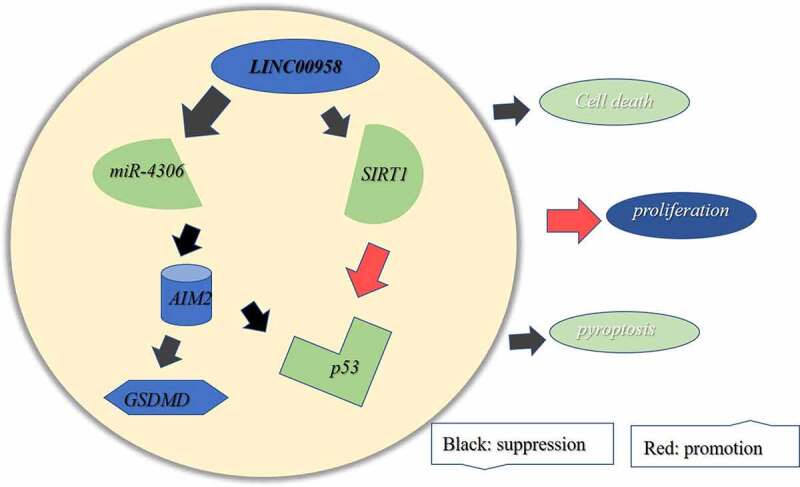

## Introduction

Oral carcinoma is the most common head and neck malignancy, mainly squamous-cell carcinoma. Together with targeted therapies, early diagnosis, especially the diagnosis of OSCC cell differentiation at different degrees, is important for the survival and prognosis of patients. At present, OSCC patients still have rather poor prognosis [1,2]. Studies have found that LncRNA, as the ceRNA of miRNA, can play an important role in cancer-related signaling pathways, thus affecting the development of cancer. LncRNAs are noncoding RNAs with length longer than 200 nucleotides [3]. The sponge effect of LncRNA on microRNA (miRNA) which participates in OSCC survival [4–6], is one of the important mechanisms of the two interactions. Some studies have demonstrated that lncRNA can regulate miRNA aggregation and biological function as competitive endogenous RNA (ceRNA) [7]. LINC00958 has been identified as oncogenes in some cancers, such as bladder cancer and OSCC [8,9], which were recognized to be as a sponge of miRNA to promote cell proliferation in some cancer cells [10–12]. It was reported that LINC00958 can competitively bind to miRNA with hepatoma-derived growth factor (HDGF) to regulate the biological functions of mRNA in hepatocellular carcinoma [10,13]. miRNAs can form a complex with Argonaute protein, which causes degradation or translation inhibition of target mRNA molecules through incomplete complementary binding with the 3ʹUTR of mRNA, thus regulating gene expression [14]. The SIRT1 belongs to class III histone deacetylase (HDAC) family, which is involved in the energy metabolism, proliferation and apoptosis of cells through deacetylation of histones and numerous non-histones (such as, p53) [15,16]. SIRT1 is highly expressed in human squamous cell carcinoma, basal cell carcinoma and other tumor tissues, which is involved in the process of cancer cell growth [17,18]. However, the study also demonstrates that SIRT1 plays a negative regulatory role in tumorigenesis. SIRT1 presents lower expression and is considered as a potential tumor suppressor in OSCC [19]. SIRT1 is considered to be involved in maintaining the integrity of epithelial cells. Hypermethylated SIRT1 occurs in OSCC patients chewing betel quid and is closely correlated with the occurrence of OSCC in patients chewing betel quid [16]. SIRT1 inhibits cell apoptosis through regulating mitochondrial apoptosis pathway in OSCC cells. SIRT1/p53 pathway could be involved in the growth of human Oral Cancer Cells [20].

P53 is the first non-histone substrate of SIRT1 to be discovered [21]. When DNA was damaged, the multiple sites in C terminal of p53 were acetylated. SIRT1 could deacetylate p53 and regulate a series of functions related to the p53 gene network [22]. The in vivo and in vitro studies showed that SIRT1 could regulate p53-mediated cell apoptosis pathway by removing the 382nd lysine acetyl group at c terminal of p53. P53 mutant commonly occurred in OSCC cells. Wild-type p53 transfected into OSCC cells could significantly inhibit cell growth.

AIM2 mediates the recruitment of apoptosis-associated speck-like protein (ASC) and recruits Caspase1, of which activation cutting substrate GSDMD could lead to cell pyroptosis. GSDMD is a member of the gasdermin proteins family with membrane perforation activity. The study demonstrates that AIM2 shows higher expression in OSCC cells and could contribute to tumorgenesis in OSCC [23]. AIM2 promotes cell survival without wild-type p53 inducement in OSCC cells [23]. The high expression of AIM2 can suppress the function of P53, which may to a large extent account for the development of OSCC. The recognition of biomarkers and the exploration for its mechanism provides essential basis for the development of detection technologies of cancers [24,25].

Bioinformatics tool has predicted that the RNA-protein interaction binding index of SIRT1 to LINC00958 ranges from 72% to 79%. LINC00958 has a large potential to bind to SIRT1. On one hand, SIRT1 may affect the expression or degradation of LINC00958; on the other hand, LINC00958 may affect the configuration, localization or epigenetic modification of SIRT1, thus affecting SIRT1 expression and activities. Besides, it is also predicted that LINC00958 may regulate AIM2 levels by sponging miR-4306. Based on these, a hypothesis was proposed that the role of LINC00958 in OSCC could be related to the involvement of miR-4306/AIM2 and SIRT1. LINC00958 could sponge miR-4306 to regulate AIM protein levels. Therefore, in the study, we aim to analyze the mechanism of LINC00958 and miR-4306 in OSCC and anticipate to elucidate the mechanism of LINC00958 in OSCC.

## Methods

### Cell lines

The cell lines, including human oral keratinocyte (HOK), human tongue squamous cell carcinoma (A-253), human oral squamous cell carcinoma (HSC-4), human tongue squamous cell carcinoma (CAL-27) and human tongue squamous cell carcinoma (SCC-4) (ATCC, America), were separately cultured in dulbecco’s modified eagle medium (DMEM) (Hyclone, America) containing 10% fetal bovine serum (Gibco, America). 100 U/mL penicillin and 100ug/mL streptomycin (Gibco, America) were added into the medium. The cells were then cultured in a constant temperature incubator of 37°C with 5% CO_2_. The medium was replaced every 2–3 days.

### qPCR

The isolation of total RNA from OSCC cells was conducted by TRIzol reagent, following which was the reverse transcription from total RNA into cDNA. After that, the expression levels of related genes were amplified by SYBR Premix Ex Taq (TaKaRa). The result was analyzed using 2^−ΔΔCt^ method. Total miRNA was extracted according to the instructions of the Express miRNA Extraction kit (Hai Gene), and the extracted concentration and purity (A260 /A280) were determined by a Microplate Reader. The miRNA was reversed into cDNA according to the instructions of the HG Taq Man miRNA c DNA Synthesis kit (Hai Gene). The reaction system (20 μL) was prepared according to the instructions of HG Taq Man miRNA qPCR. RNU6B was used as the internal reference. The relative expression of miRNA-4306 was calculated using 2^−ΔΔCt^ method [26].

### Western blotting assay

After trypsin digestion, the cells were centrifuged at 800 g at 4°C for 3 min to collect the cells. RIPA lysis buffer (PH 7.4, containing 1 mM PMSF) was used to lyse cells. Centrifuge at 12,000 g 4°C for 10 min and transfer the supernatant to a new centrifuge tube. Protease inhibitor PMSF was added to cell lysate (PPLYGEN, Beijing, China). The protein concentrations were detected by BCA method. The proteins were separated by SDS-PAGE electrophoresis. Then, proteins were transferred onto PVDF membranes, which then were blocked by skim milk powder prepared with 5% TBS+Tween (TBST) buffer solution (PH7.5, containing 0.05% Tween) at room temperature for 1 h. The PVDF membrane was put into the hybridization box and a primary antibody of the labeled target band was added. The PVDF membranes, which were pre-incubated overnight at 4°C, were removed and washed with TBST buffer solution for 3 times, with 5 min each. The membranes were then transferred to the secondary antibody hybridization box and incubated at room temperature for 1 h. ECL luminescent solution was added to the membrane to develop color (Focus on life science, Beijing, China).

### Plasmid transfection

HSC4 cells were seeded into a six-well plate to regulate cell growth to logarithmic phase. Cells were transfected with LINC00958 knockdown plasmid (ShRNA-LINC00958, GenePharma, Shanghai, China) and its control plasmids (ShRNA-NC) through Lipofectamine 2000 (Invitrogen, USA) following manufacturer’s protocol. After 48 h, the transfection efficacy was detected through qPCR.

### Lentiviral transfection

HSC4 cells were seeded into a six-well plate to regulate cell growth to logarithm phase. The cells were transfected with lentiviral when the growth density of cells reached 60%~70% (Cyagen Biosciences, Guangzhou, China). The medium size and amount of virus recommended for viral infection of cells according to the lentivirus instructions. The virus solution of control group (the empty vector) and the virus group were diluted with the non-dual antibody medium, and the final concentration of 8 μg/m L polybrene was added, respectively. The final volume of each well in the six-well plate was 1 m L. Western blot or qPCR was used to detect the expression LINC00958 or SIRT1 after transfection with lentiviral.

### Luciferase reporter gene reporter assay

MiR-4306 mimic and its control miR-4306 NC were purchased from (Shanghai, China). 50 nmol/L miR-4306 mimic or NC and 100 nmol/L wild or mutant dual luciferase reporter vector (RiboBio, Guangzhou, China) were co-transfected into 293 T cells (1.5×10^4^) using Attractene Transfection Reagent (QIAGEN, Germany) according to manufacturer’s instructions. 48 h after transfection, luciferase activities were detected by double luciferase detection system (Progema).

### CCK-8 assay

At logarithmic growth stage, the cells were seeded into 96-well plates. After transfection of 48 h,10 µL CCK-8 solution was added to each well, and the cells were further cultured for 2 h after shaking (CCK8, Beyotime, Shanghai, China). The absorbance value at 490 nm was detected by a microplate reader.

### Immunofluorescence (IF) assay

After transfection of 48 h, the cells were fixed with 4% paraformaldehyde and sealed with 1% BSA for 30 min. The primary antibody (Santa Cruz, USA) was added and placed in a wet box overnight at 4°C. On the next day, the primary antibody was discarded, and FITC-labeled secondary antibody (Santa Cruz, USA) was added to the sections and incubated for 30 min. Then, the cells were incubated with DAPI for 5 min, sealed with anti-fluorescence quencher, and observed under a confocal microscope.

### Tunel staining

Cells were fixed using 4% paraformaldehyde and then washed once with PBS. PBS containing 0.3% Triton x-100 was added to the sections and incubated at room temperature for 5 min. The apoptotic cells were stained using TUNEL kit according to the protocol (Beyotime, Shanghai, China). The process was as follows: The cell sections were washed once with PBS. 50 μL biotin-labeled solution was added to the section and incubated for 60 min at 37°C in the dark. After washing once with PBS, 0.1–0.3 ml stop solution was dropped to sections, and incubated at room temperature for 10 min. 50 μL streptavidin-HRP working fluid was added to the sections and incubated at room temperature for 30 min. Then, 0.2–0.5 ml DAB solution was added and incubate at room temperature for 30 min. The nuclei were stained with hematoxylin.

### RNA- binding protein immunoprecipitation (RIP)

Cells were washed using PBS and then lysed with RIP Lysis buffer (200 µl) and 10% protease inhibitor on ice for 10 min. The supernatant was collected, the protein concentration of which was measured by BCA kit protein. 30–40 g protein was taken as Input group to detect GADPH and SIRT1 expression. 1 mg protein was taken as IgG or SRIT1 group, and 10 µl immunomagnetic beads were added (Abcam, America). Then, IgG or anti-SRIT1 antibody (2 µg) was added to incubate for 14 h at 4°C. Immunomagnetic beads (30 µL) was added to incubate for 2 h. After centrifugation at 2500 g for 30 s. RIP buffer (400 µL) was used to suspend beads, which then were washed with PBS for 3 times. TRIzol was used to extract total RNA to detect the expression of LINC00958 by qPCR. The experiment was repeated for 3 times.

### Statistical analysis

Prism 7.0 software was used to perform statistical analysis. Measurement data were tested for normality. Data conforming to the normal distribution were expressed as mean ±standard deviation (SD). The comparison among the groups was conducted by ANOVA analysis, and then the pairwise comparison was conducted by LSD-t test. P < 0.05 was considered statistically significant.

## Results

### The expression of LINC00958, AIM2, GSDMD, and TP53 in oral cancer cells

**Figure 1.**
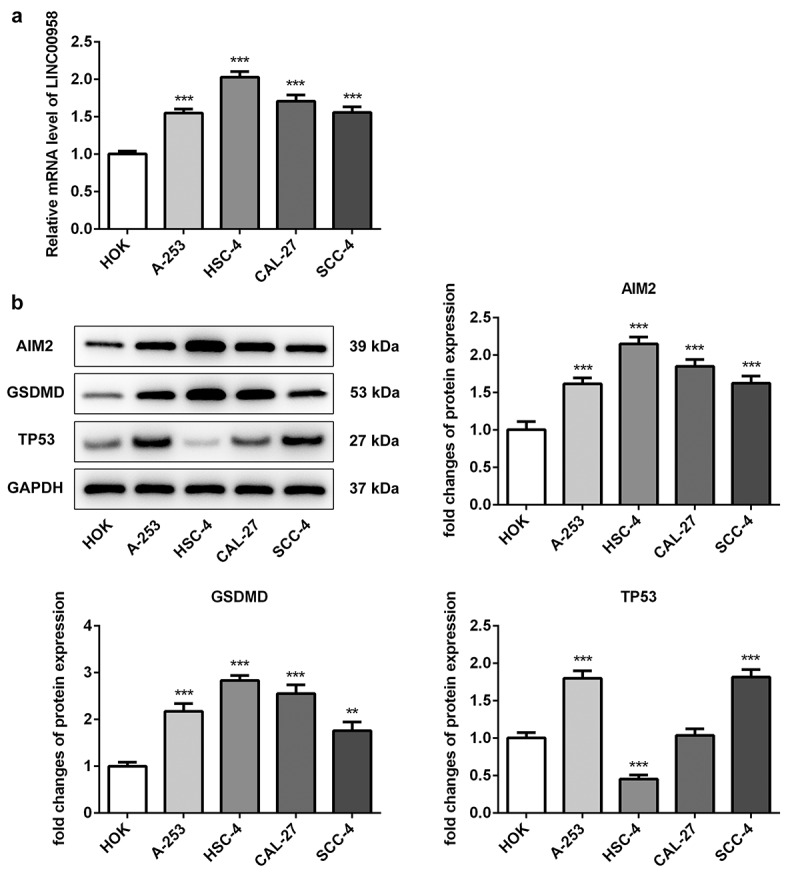
A LINC00958 expression was analyzed in different oral cancer cell lines through qPCR. B Western blot analyzed the expression of AIM2, GSDMD and p53 in different oral cancer cell lines. HOK: normal oral epithelial cells. A-253: human salivary gland tumor cells. HSC-4: Human OSCC cells.CAL-27, SCC4: human tongue squamous cell. Data were shown as mean±SD

To investigate the regulatory role of LINC00958 in OSCC, different oral cancer cell lines were used to analyze the expression of LINC00958, AIM2, GSDMD and TP53. The results showed that LINC00958 presented higher expression in A-253, HSC-4, CAL-27 and SCC-4 cells than HOK cells. Moreover, LINC00958 expression seemed to be higher in HSC-4 cells compared to other oral cancer cell lines (Figure 1(a)). Thus, we chose HSC-4 cells for further experiments. Besides, AIM2 and GSDMD showed higher expression in oral cancer cells than HOK cells, especially in HSC-4 cells (Figure 1(b)). P53 expression was significantly downregulated in HSC-4 cells than HOK cells. The result was similar to those in the previous study [27].

### LINC00958 knockdown significantly reduced OSCC cell proliferation and promoted cell death

**Figure 2:**
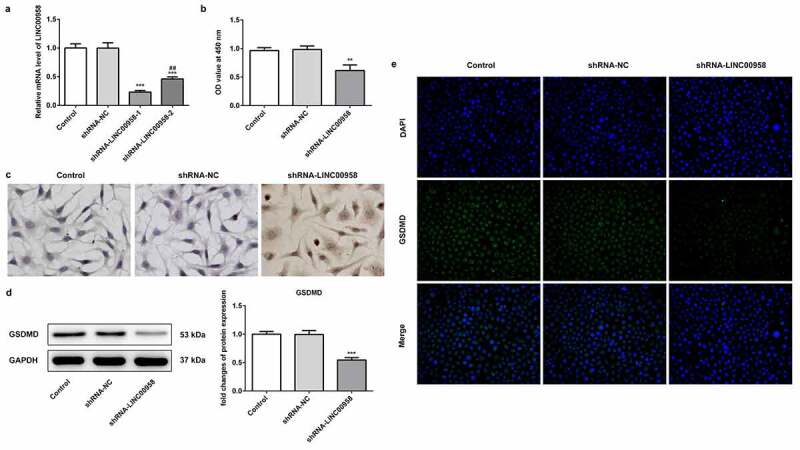
A LINC00958 interference plasmids (ShRNA-LINC00958-1 or ShRNA-LINC00958-2) significantly reduced endogenous LINC00958 expression. B CCK8 assay analyzed the cell proliferation in HSC4 cells. C Tunel staining analyzed the cell apoptosis in HSC4 cells. D. Western blot showed that LINC00958-1 knockdown reduced GSDMD expression. E. IF (immunofluorescence) analyzed GSDMD expression. ShRNA-LINC00958: LINC00958-1 knockdown plasmids were transfected into HSC4 cells. Data were shown as mean±SD. ***p < 0.001 compared with ShRNA-NC. ^##^p < 0.01 compared with ShRNA LINC00958-1

To reveal the role of LINC00958 in OSCC cell progression, LINC00958 interference plasmids (ShRNA-LINC00958-1 or ShRNA-LINC00958-2) were separately used to decrease endogenous LINC00958 expression in HSC4 cells. The results showed that ShRNA-LINC00958-1 has better inhibitory effects on LINC00958-1 expression than ShRNA-LINC00958-2 (Figure 2(a)). Thus, ShRNA-LINC00958-1 was utilized to perform further experiments. Then, we analyzed the effects of ShRNA-LINC00958-1 on proliferation and apoptosis of HSC4 cells (Figure 2(b-c)). The results showed that ShRNA-LINC00958 silencing suppressed cell proliferation and promoted cell apoptosis compared to control group. Western blot and IF showed that LINC00958 knockdown significantly reduced GSDMD levels (Figure 2(d-e)).

### LINC00958 regulated apoptosis, inflammasome, and autophagy-related pathway

**Figure 3:**
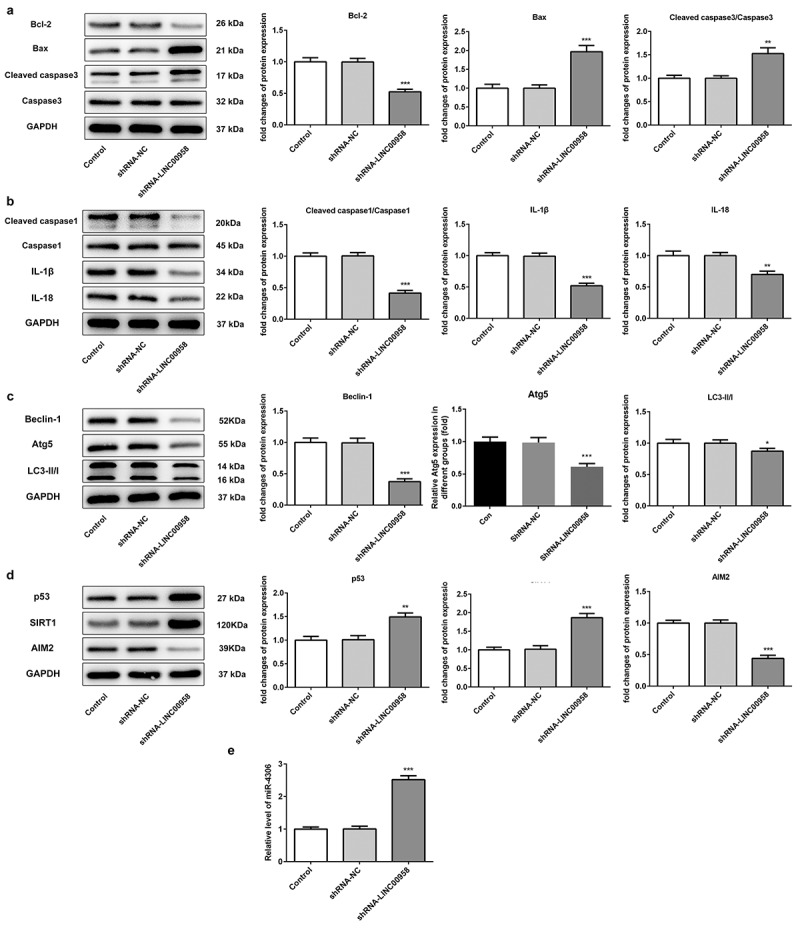
A ShRNA-LINC00958 regulated OSCC cell apoptosis pathway. B ShRNA-LINC00958 reduced OSCC cell pyroptosis. C ShRNA-LINC00958 decreased OCSS cell autophagy. D ShRNA-LINC00958 upregulated p53 and SIRT1 levels, reducing AIM2 expression. E ShRNA-LINC00958 increased miR-4306 expression. Data was shown as mean±SD. ***p < 0.001 or **p < 0.01 compared with ShRNA-NC

Next, we wondered to know whether LINC00958 was involved in cell apoptosis, pyroptosis, and autophagy. LINC00958 silencing markedly increased the levels of pro-apoptosis proteins Bax and cleaved-caspase-3, accompanied by decreased anti-apoptosis protein Bcl-2 expression as contrast to control group, which could lead to OSCC cell apoptosis (Figure 3(a)). By analyzing the expression of inflammasome-mediated proteins, cleaved caspase-1, IL-1β, IL-18 were significantly reduced by LINC00958 silencing in HSC cells, which implied that LINC00958 inhibited the activation of inflammasome and reduced the level of pyroptosis (Figure 3(b)). In addition, the ratio of LC3-II/LC3-I was significantly reduced by LINC00958 silencing when compared with control group (Figure 3(c)). Furthermore, autophagy-related proteins, Beclin-1 and Atg5 were also significantly decreased. The results implied that autophagy levels could be reduced by LINC00958 silencing. All in all, the downregulation of LINC00958 significantly increased OSCC cell apoptosis, along with reduced cell pyroptosis and autophagy. We further found that LINC00958 silencing significantly upregulated p53 and SIRT1 levels, as well as reduced AIM2 levels (Figure 3(d)). qPCR analysis showed that miR-4306 was markedly increased by decreasing endogenous LINC00958 expression (Figure 3(e)). Thus, we supposed that the downregulation of LINC00958 could not only decrease cell pyroptosis by inhibiting AIM2 expression (for tumor survival) but also reduced the inhibitory effect of p53, thereby increasing cell apoptosis and decreasing autophagy and proliferation. Overall, the latter effect is more obvious. Therefore, LINC00958 downregulation presented the inhibitory effects on cell proliferation.

### LINC00958 interacted with SIRT1

**Figure 4:**
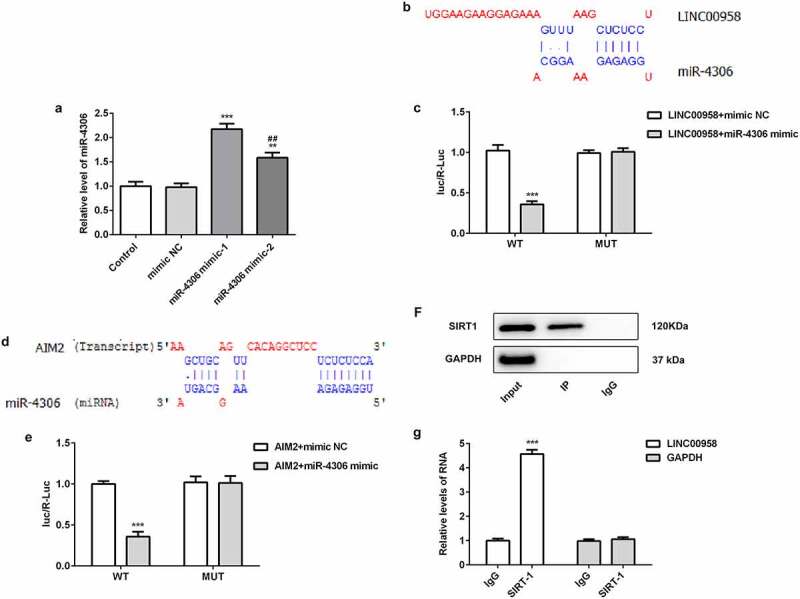
A miR-4306 mimic upregulated endogeneous miR-4306 levels. ***p < 0.001 or **p < 0.01 compared with mimic NC. ^##^p < 0.01 compared with miR-4306 mimic-1

In order to analyze the association of LINC00958 and SIRT1, different miR-4306 mimic plasmids were separately transfected into OSCC cells to analyze the effects of miR-4306 mimic on upregulating endogenous miR-4306 levels (Figure 4(a)). The results showed that miR-4306 mimic-1 has better effects than miR-4306 mimic-2. Then, the bioinformatics analysis found that miR-4306 could bind to the 3ʹUTR of LINC00958 (Figure 4(b)). Luciferase gene reporter assay showed that fluorescent intensity was significantly reduced in miR-4306 mimic and wild-type LINC00958 treatment than mutant LINC00958, which indicated that miR-4306 bound to the 3ʹUTR of LINC00958 (Figure 4(c)). The association of AIM2 and miR-4306 was also analyzed through bioinformatics analysis and luciferase gene reporter assay (Figure 4(d-e)). The result showed that miR-4306 bound to the 3ʹUTR of AIM2. Then, we analyzed whether LINC00958 interacted with SIRT1 using RIP assay. Anti-SIRT1 antibody was used to pull down SIRT1 complex. The analysis of immunoprecipitation by qPCR showed that LINC00958 was enriched, which implied that SIRT1 interacted with LINC00958 (Figure 4(f-g)).

B-C Bioinformatics analysis and luciferase gene reporter assay showed that miR-4306 bound to the 3ʹUTR of LINC00958. D-E. Bioinformatics analysis and luciferase gene reporter assay showed that miR-4306 bound to the 3ʹUTR of AIM2. **p < 0.01 compared with WT-LINC00958 + mimic NC. F-G. RNA-binding protein immunoprecipitation (RIP) showed that Anti-SIRT1 antibody significantly enriched LINC00958 in immunoprecipitation. Data was shown as mean±SD. ***p < 0.001 compared with WT-AIM +mimic NC.

### LINC00958 regulated cell survival through AIM2 and SIRT1

**Figure 5:**
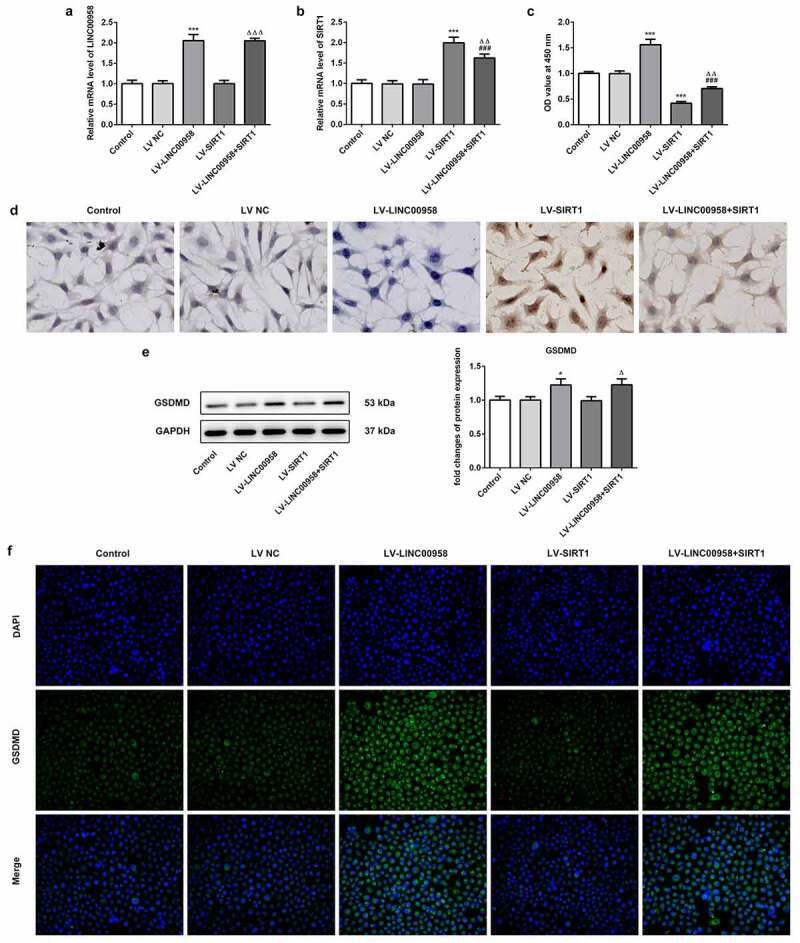
A. qPCR analyzed the expression of LINC00958 and SIRT1 after transfecting Lentiviral vector overexpressing LINC00958 or SIRT1 into HSC4 cells. B the effect of LINC00958 on SIRT1 mRNA. C. Cell proliferation abilities were detected by CCK8 assay. D. Tunel staining evaluated cell death rate. E-F LV-LINC00958 upregulated GSDMD expression detected by WB and IF assay. LV-LINC00958: Lentiviral vector overexpressing LINC00958. LV-SIRT1: Lentiviral vector overexpressing SIRT1. LV-LINC00958+ SIRT1: simultaneously overexpressing LINC00958 and SIRT1. Data was shown as mean±SD. ***p < 0.001 or *p < 0.05 compared with LV-NC. ^ΔΔΔ^ p < 0.001, ^ΔΔ^p<0.01 or ^Δ^p<0.05 compared with LV-SIRT. ^###^ p < 0.001 compared with LV-LINC00958

To reveal how LINC00958 affect OSCC cells survival, lentiviral vectors simultaneously overexpressing LINC00958 and SIRT1 (being expressed as LV-LINC00958+ SIRT1), or alone overexpressing LINC00958 or SIRT1 (separately being expressed as LV-LINC00958 and LV-SIRT1) were transfected into HSC4 cells, qPCR was utilized to analyze the transfection efficacy. When LINC00958 was inserted into lentiviral vector alone, the expression levels after transfection were basically the same as that of digene insertion (Figure 5(a)). LV-LINC00958 significantly increased cell proliferation and decreased death rate through the analysis of CCK8 assay and Tunel staining while LV-SIRT1 presented adverse effects when compared with control group (Figure 5(c-d)). SIRT1 overexpression notably reversed the effects of LINC00958 on cell proliferation and death. LV-LINC00958 increased GSDMD expression. However, LV-SIRT1 did not exhibit significant effects on GSDMD expression (Figure 5(e-f)). Therefore, LV-LINC00958 could affect cell survival partly depending on SIRT1 pathway. Additionally, LINC00958 induced an increase in GSDMD protein expression independent on GSDMD, which was considered to be related to miR-4306/AIM2.

### LINC00958 regulated cell death and autophagy through AIM2 and SIRT1

**Figure 6.**
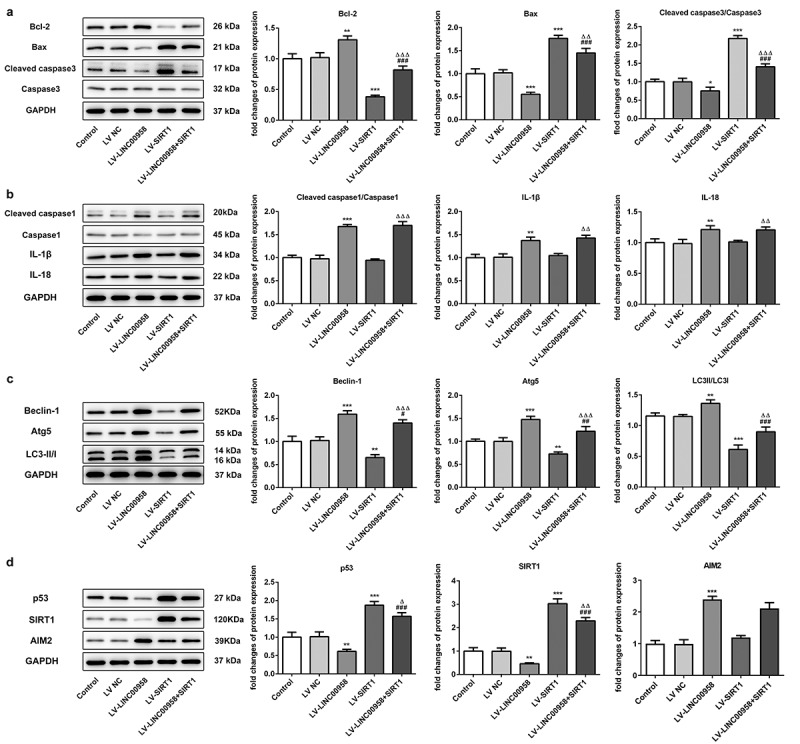
A. LINC00958 and SIRT1 regulated mitochondrial-mediated apoptosis pathway. B LINC00958 regulated inflammasome-mediated pyroptosis-related proteins. C. LINC00958 and SIRT1 regulated autophagy-related proteins. D p53, SIRT1 and AIM2 levels were affected by LINC00958 and SIRT1. Data was shown as mean±SD. ***p < 0.001 or **p < 0.01 compared with LV-NC. ^ΔΔΔ^ p < 0.001, ^ΔΔ^p<0.01 or ^Δ^p<0.05 compared with LV-SIRT. ^###^ p < 0.001, ^##^ p < 0.01 or ^#^p < 0.05 compared with LV-LINC00958

To further explore how LINC00958 affected cell apoptosis, pyroptosis and autophagy, the expression of mitochondrial-mediated apoptosis-related protein was analyzed after overexpressing LINC00958 or SIRT1 in HSC4 cells (Figure 6(a)). We found that LV-LINC00958 upregulated anti-apoptosis protein Bcl-2 and downregulated pro-apoptosis proteins Bax, c-casp-3 while LV-SIRT1 presented adverse effects. Inflammasome-mediated pyroptosis-related proteins c-casp-1, IL-1β and IL-18 were also significantly increased by LINC00958 while LV-SIRT1 presented no effects (Figure 6(b)). Overexpressing SIRT1 significantly blocked these effects of LINC00958 on cell apoptosis. In addition, autophagy-related proteins Beclin-1 and Atg5 were markedly increased by overexpressing endogenous LINC00958 levels, along with the increased ratio of LC3-II/LC3-I (Figure 6(c)). However, LV-SIRT1 showed adverse effects, which could be reversed by LV-LINC00958 treatment. Therefore, LINC00958 could decrease cell apoptosis and increase autophagy through regulating these proteins via SIRT1. However, LINC00958 decreased cell pyroptosis-related proteins independent on SIRT1. Then, we found that LINC00958 overexpression reduced p53 and SIRT1 levels, accompanied by the increases in AIM2 levels (Figure 6(d)). SIRT1 overexpression significantly increased p53 levels and reversed the effects of LINC00958 on p53. Thus, LINC00958 regulated AIM2/GSDMD independent on SIRT1 and reduced p53 levels through regulating SIRT1 and AIM2 expression.

## Discussion

In our work, OSCC cells displayed increased LINC00958 levels compared with HOK cells. We also found that the levels of LINC00958, AIM2, GSDMD and TP53 seem to be higher when relative to other OSCC cells while TP53 showed less expression. Furthermore, this study showed the role of LINC00958 in regulating AIM2/GSDMD, cleaved caspase1, IL-1β, and IL-18 levels, which indicated the close association of LINC00958 with AIM-mediated pyroptosis pathway. Moreover, no significant change was found in their expression after SIRT1 overexpression. Thus, we can conclude that the regulation of LINC00958 for AIM-mediated pyroptosis pathway is irrelevant to SIRT1.

AIM2 was investigated to regulate p53 [23,28] andSIRT1 was validated to mediate the regulation of LINC00958 for p53. Taken together, p53 levels were regulated by LINC00958 mediated by AIM2 and SIRT1. Additionally, AIM2 was subjected to the sponge of miR-4306, the levels of which were regulated by LINC00958. As a result, LINC00958/miR-4306 regulated pyroptosis and p53 levels by AIM, and affected p53 expression via SIRT1, consequently promoting tumor survival through enhancing cell proliferation and reducing cell death.

By silencing or overexpressing LINC00958, the proliferation, apoptosis-related autophagy-related and pyroptosis-related proteins were markedly affected, which was considered to possibly be related to p53. A study has shown that lncRNA is involved in regulating different signaling pathways including p53 signal and mediating malignant progression in oral submucous fibrosis through bioinformatics analysis [29]. Besides, miRNA has been reported to be implicated in regulating p53 expression and OSCC cell proliferation [29]. The effects of LINC00958 on promoting OSCC cell survival possibly is finally mediated mainly through reducing p53 levels. p53 inactivation and mutant are the risk factors which facilitate head and neck squamous cell carcinoma progression [30,31]. According to the analysis of differential expression genes in OSCC, we found that these genes are tightly correlated with p53 signaling pathway [32],which could regulate cell proliferation, apoptosis and autophagy [33,34]. Furthermore, the OSCC cell apoptotic levels could be significantly inhibited through targeting p53 [35]. Increased levels of p53 in the cytoplasm are involved in inhibiting autophagy [36]. A study suggests that p53 could regulate expression of Bcl-2 family protein and increase permeability of mitochondrial outer membrane to induce apoptosis [37,38]. Therefore, decreased p53 levels could contribute to the suppression of cell death and increased autophagy in OSCC cells. In our study, SIRT1 overexpression significantly upregulated p53 levels. A study has shown that SIRT1 not only deacetylated p53 to reduce p53 activities but also regulated p53 levels.

Our study also showed that GSDMD presented higher expression in OSCC cells than HOK cells and LINC00958 significantly upregulated GSDMD levels. Besides, cleaved caspase-1 and IL-1β protein expression was also increased. GSDMD punch holes in the membrane, causing cell osmotic pressure change and pyroptosis [39]. GSDMD was also involved in inducing the release of inflammatory factors such as IL-1β and IL-18, which can promote the growth of gastric cancer cells and accelerate the formation of gastric tumors through immunosuppression [40,41]. Besides, mitochondrial damage is considered to be a key event in GSDMD inducing cells into pyroptosis [42]. The interactions between autophagy and apoptosis processes have been widely discussed by many reports. On one hand, during the process of autophagy, the damaged mitochondria will be captured, degraded and then eliminated so as to prevent the cells from apoptosis. On the other hand, the cell suicide mechanism will initiate upon the failure of autophagy to rescue these cells. Activated caspase destroys the functions of several important autophagy proteins, leading to the paralyzation of the autophagy process, termination of the cell self-defense functions, and the acceleration of cell death. Therefore, increased autophagy by LINC00958 could suppress apoptosis process.

In summary, LINC00958 led to a decrease in p53 levels via AIM2 mediated by miR-4306 and SIRT1, and activated AIM2 caused cell pyroptosis pathway via miR-4306, consequently promoting cell proliferation and autophagy, as well as reducing cell apoptosis. The study provides new targets for developing novel drugs for the therapy of OSCC.

## Conclusion

In OSCC cells, LINC00958 has an effect on cell proliferation, apoptosis and autophagy via two pathways: One is that LINC00958 greatly exerts pro-cancer effects possibly via p53 mediated by SIRT1 and AIM2. The other is that LINC00958 also could promotes cell pyroptosis via AIM2/GSDMD depending on miR-4306. Totally, the promoting cancer effects of LINC00958 take the leading position. The study opens a window for understanding the pathomechanism of OSCC, recognizes some potential targets for the diagnosis and therapy of OSCC and lays the foundation of future studies of in vivo.

## Data Availability

The datasets used and/or analyzed during the current study are available from the corresponding author on reasonable request.

## Highlights

1. The role of LINC00958 in OSCC is closely related to cell proliferation, autophagy, and pyroptosis.

2. The regulatory mechanism of LINC00958 in OSCC was mediated by AIM2 and SIRT1.

3. LINC00958 not only regulated p53 by both SIRT1 and AIM2 but also regulated AIM2/GSDMD by miR-4306.
